# Application of musculoskeletal ultrasound in endemic skeletal fluorosis: a comparative study of water-borne and brick-tea–borne subtypes

**DOI:** 10.3389/fpubh.2026.1877861

**Published:** 2026-09-09

**Authors:** Xiaoli Hu, Siyu Zhang, Yuanyuan Chen, Fangying Liu

**Affiliations:** 1 Ultrasound Center, Affiliated Hospital of Guizhou Medical University, Guiyang, China; 2 School of Medical Imaging, Guizhou Medical University, Guiyang, China

**Keywords:** digital radiography, endemic fluorosis, interosseous membrane, MSKUS, musculoskeletal ultrasound, skeletal fluorosis

## Abstract

**Background:**

Endemic skeletal fluorosis is a chronic disorder caused by prolonged excessive fluoride intake and is characterized by skeletal hyperostosis, periarticular soft-tissue degeneration, and joint dysfunction. Digital radiography (DR) is the conventional diagnostic modality, but it has limited sensitivity for early soft-tissue changes and provides little dynamic information. Musculoskeletal ultrasound (MSKUS) may address these limitations, yet comparative evidence across major fluorosis subtypes remains limited.

**Objective:**

To characterize MSKUS findings in endemic skeletal fluorosis and compare the complementary value of MSKUS and DR in water-borne and brick-tea-borne disease cohorts.

**Methods:**

This retrospective regional comparative study included 246 patients from Jishan County, a predominantly water-borne fluorosis area (*n* = 132), and Ruoergai County, a predominantly brick-tea-borne fluorosis area (*n* = 114). DR was performed at baseline and 3 months after fluoride-exposure reduction. MSKUS was performed only at the three-month follow-up and assessed elbow and knee abnormalities and forearm interosseous membrane (IOM) thickness. Because baseline MSKUS was unavailable, ultrasound findings were analyzed cross-sectionally.

**Results:**

Patients from Jishan County were older (64.73 ± 7.73 vs. 59.35 ± 9.52 years) and more often female (58.33% vs. 44.83%) than those from Ruoergai County (both *p* < 0.05). Ruoergai patients had higher MSKUS detection rates of humeroulnar joint calcification (78.07% vs. 15.15%), humeroradial joint calcification (48.25% vs. 3.03%), elbow effusion (32.46% vs. 0.76%), and suprapatellar bursa effusion (39.47% vs. 12.12%; all *p* < 0.05). Forearm IOM thickness in pronation was also greater in Ruoergai patients (2.02 ± 0.54 vs. 1.83 ± 0.49 mm; *p* < 0.05). DR showed a higher observed detection rate for radial crest enlargement than MSKUS (94.3% vs. 50.0%), whereas MSKUS showed a higher observed detection rate for radius-ulna IOM calcification (73.6% vs. 15.9%). Fluorosis severity grades differed between baseline and follow-up (*p* < 0.05), but this short interval precludes inference of structural reversal.

**Conclusion:**

MSKUS and DR provide complementary information in endemic skeletal fluorosis. MSKUS may improve detection of superficial soft-tissue calcification and effusion, whereas DR remains important for osseous abnormalities. Prospective multicenter studies with standardized ultrasound protocols and paired baseline and follow-up MSKUS are warranted.

## 1 Introduction

Endemic skeletal fluorosis is a chronic metabolic bone disease caused by prolonged excessive fluoride exposure. Chronic fluoride accumulation in bones and joints may disturb bone mineral metabolism and lead to osteosclerosis, ligamentous calcification, joint stiffness, restricted mobility, and functional impairment. The disease remains a substantial public health concern in fluoride-endemic regions worldwide, particularly in rural areas of Asia and Africa, including parts of India and China (1, 2). At the molecular level, excessive fluoride can be incorporated into bone mineral, where fluoride deposition and fluoroapatite crystal formation may disrupt normal bone remodeling and contribute to abnormal mineralization, trabecular thickening, sclerosis, and altered biomechanical properties of bone tissue (3). Endemic skeletal fluorosis remains a serious public health concern in affected regions, particularly where long-term exposure to high-fluoride drinking water or other fluoride-rich sources contributes to progressive skeletal involvement, joint stiffness, ligament calcification, functional impairment, and disability. Given the limited availability of specific treatment options, early recognition, improved diagnostic assessment, and timely reduction of fluoride exposure are essential for reducing disease burden (1).

Conventional radiography, including digital radiography (DR), has long played an important role in the imaging evaluation of skeletal fluorosis. Radiographic features of endemic skeletal fluorosis include osteosclerosis, osteopenia, growth lines, diaphyseal widening, and calcification or ossification of ligamentous, tendinous, muscular attachment sites and interosseous membranes (4). Nevertheless, radiography mainly depicts mineralized structural abnormalities and is less informative for non-ossified superficial soft-tissue changes, joint effusion, and real-time dynamic assessment. These limitations support the use of musculoskeletal ultrasound as a complementary modality for evaluating periarticular soft-tissue involvement. Musculoskeletal ultrasound (MSKUS) has gained increasing recognition as a complementary imaging tool owing to its non-invasive nature, real-time dynamic imaging capacity, and high resolution for soft-tissue structures. Evidence suggests that MSKUS can detect soft-tissue calcifications, synovial hypertrophy, and joint effusions in real time, making it particularly advantageous in resource-limited settings prevalent in endemic fluorosis regions (5–7).

Among the exposure-related forms of endemic skeletal fluorosis in China, water-borne and brick-tea–borne fluorosis are the two forms compared in the present study. The water-borne subtype is mainly associated with long-term consumption of drinking water containing excessive fluoride (8, 9), whereas the brick-tea–borne subtype is related to habitual intake of fluoride-rich brick tea in some Tibetan and other minority populations (10–12). Previous studies have shown that brick tea is commonly produced from mature leaves and stems of the tea plant, which may contain higher fluoride concentrations than ordinary tea, and long-term consumption of brick tea can contribute substantially to total fluoride intake (10–12). In adult populations from brick-tea–drinking areas, skeletal fluorosis has been associated with osteosclerosis, arthropathy, interosseous membrane ossification, tendon attachment calcification, articular degeneration, and functional impairment (10, 11). However, whether differences in musculoskeletal imaging phenotypes are attributable to fluoride source itself or to accompanying regional, dietary, environmental, and demographic factors remains uncertain. Therefore, comparative imaging data between water-borne and brick-tea–borne endemic fluorosis remain needed.

To address this gap, the present study enrolled patients from Jishan County, Shanxi Province (a water-borne fluorosis region) and Ruoergai County, Sichuan Province (a brick-tea–borne fluorosis region). Using both MSKUS and DR, we compared the observed imaging profiles of joint calcification, joint effusion, and IOM abnormalities between these two regional cohorts. Because MSKUS was performed only at the three-month follow-up, ultrasound findings were analyzed cross-sectionally rather than longitudinally. The aim of this study was to describe the MSKUS manifestations of endemic skeletal fluorosis and to explore the complementary roles of MSKUS and DR in characterizing musculoskeletal involvement.

## 2 Materials and methods

### 2.1 Study design and patient selection

**Figure 1:**
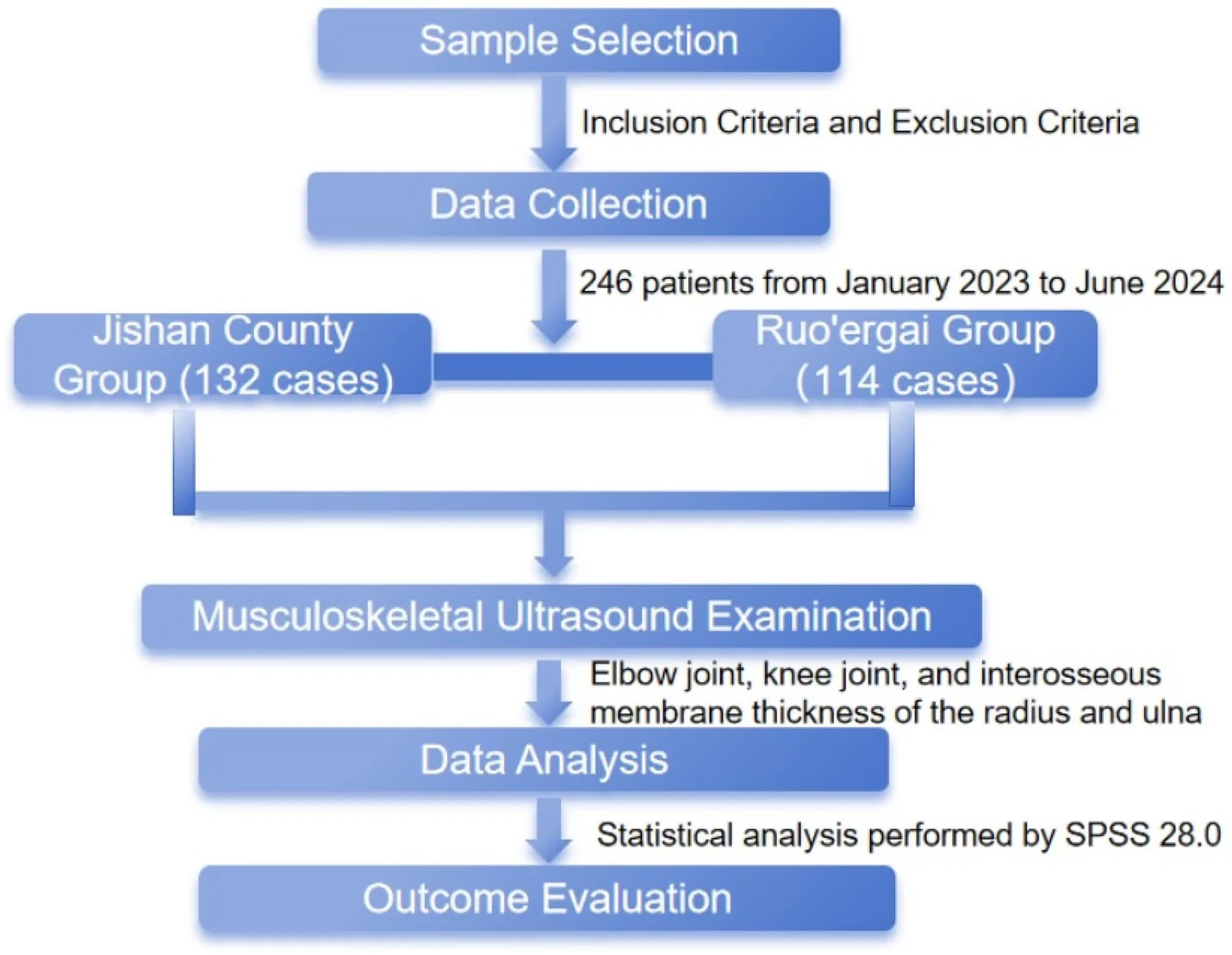
Flowchart of patient recruitment and study procedures.

This retrospective study reviewed clinical data from 246 patients diagnosed with endemic skeletal fluorosis based on epidemiological surveys conducted between January 2023 and June 2024. The patient recruitment process, cohort assignment, and imaging assessment timeline are illustrated in Figure 1. Patients were allocated to two groups according to geographic origin and fluoride source: the Jishan County group (water-borne fluorosis, *n* = 132) and the Ruoergai group (brick-tea–borne fluorosis, *n* = 114). DR examinations were performed at baseline and 3 months after fluoride-exposure reduction, whereas MSKUS examinations were performed only at the three-month follow-up; therefore, MSKUS findings were analyzed as cross-sectional follow-up imaging findings rather than longitudinal ultrasound changes. The study protocol was approved by the institutional ethics committee (2023–189), and written informed consent was obtained from all participants.

Inclusion criteria were as follows: (1) continuous long-term residence in Jishan County or Ruoergai County without extended stays elsewhere; (2) diagnosis of endemic skeletal fluorosis confirmed according to the Chinese national standard WS/T 192–2021 (13); (3) imaging evidence (CT, MRI, or radiography) demonstrating joint dysfunction or neurological involvement consistent with skeletal fluorosis; and (4) availability of complete MSKUS data. Patients were excluded if they refused examination or had incomplete medical records; if they carried a diagnosis of primary osteoporosis, osteomalacia, renal osteodystrophy, or osteoblastic metastatic disease; or if they had significant comorbidities—including hepatic or renal insufficiency, systemic infection, or malignancy—that could confound imaging findings or interfere with follow-up.

### 2.2 Imaging procedures

#### 2.2.1 Musculoskeletal ultrasound

Ultrasound examinations in the Ruoergai group were performed using a GE Vivid E7 system (probe frequency 9L), while a Neusoft NeuEcho 10 system (probe frequency L12-4) was used for the Jishan County group. Examinations proceeded in a standardized sequence: upper-limb elbow joint followed by lower-limb knee joint. Forearm IOM measurements were obtained with the forearm in pronation, radial crest measurements with the forearm in supination, and all remaining assessments were performed in the supine position. To reduce measurement variability, all examinations followed a predefined standardized scanning protocol, including fixed patient positioning, anatomical landmarks, scanning planes, and measurement sites. Forearm IOM thickness was measured at the thickest transverse section where the radius, ulna, and interosseous membrane were clearly visualized. Each IOM thickness measurement was repeated three times at the predefined site, and the mean value was used for statistical analysis. However, no formal inter-equipment calibration, phantom-based reproducibility assessment, or cross-platform validation was performed between the two ultrasound systems. No formal intra-observer or inter-observer reliability analysis was performed for MSKUS measurements; measurement consistency was addressed by using standardized scanning planes, predefined anatomical landmarks, and triplicate measurements averaged for analysis.

Elbow joint assessment encompassed the anterior compartment (transverse scanning with the forearm supinated), the pronator teres and its radial attachment (longitudinal and transverse scanning from the anterior elbow), the anterior joint recess (sagittal evaluation of the coronoid fossa for effusion between the fat pad and humerus), the lateral compartment (long- and short-axis views of the common extensor tendon at the lateral epicondyle), the humeroradial joint (dynamic scanning during passive forearm pronation and supination), the medial compartment (common flexor tendon and medial collateral ligament in long-axis view), the posterior compartment (triceps tendon evaluated with the elbow flexed to 90°), and IOM thickness measurement (forearm in pronation with shoulder abducted 90°; active finger flexion and extension facilitated clear visualization of the IOM).

Knee joint assessment included the quadriceps tendon (longitudinal scanning superior to the patella with the knee flexed 20°), the patellar tendon (longitudinal and transverse planes from origin to insertion), the medial collateral ligament and pes anserine tendons (knee flexed 20°–30°, leg externally rotated), the iliotibial band (knee flexed 20°–30°, leg internally rotated), the lateral collateral ligament (long- and short-axis views superior to the fibular head), and leg IOM thickness (measured at the mid-lateral leg when the membrane was clearly delineated).

Recorded findings included the presence and anatomical location of calcifications in the humeroulnar joint, humeroradial joint, common extensor tendon, common flexor tendon, ulnar collateral ligament (UCL), radial collateral ligament, quadriceps tendon, medial collateral ligament, pes anserine tendon, iliotibial band, and patellar tendon, as well as detection of elbow and suprapatellar bursa effusions. Forearm IOM thickness (pronation) and leg IOM thickness at the mid-shaft were recorded quantitatively.

#### 2.2.2 Digital radiography

Standardized DR examination was performed in all patients before and 3 months after intervention. Assessed parameters included radial crest enlargement (evaluated on anteroposterior and lateral elbow views), radius-ulna IOM calcification (anteroposterior forearm view), tibia-fibula IOM calcification (anteroposterior leg view), cortical loosening at the pronator attachment (elbow-focused views), and soleus tendon calcification (posterior lower-extremity views). All images were independently reviewed by two experienced radiologists; discrepancies were resolved by consensus.

### 2.3 Assessment of fluorosis severity and intervention

Fluorosis severity was graded according to WS/T 192–2021 criteria (13) at baseline and 3 months after intervention. Severity grading was primarily based on clinical assessment combined with DR findings according to WS/T 192–2021. MSKUS findings were not used for severity reclassification. The exposure-reduction and follow-up procedures were implemented within the same field-management framework of the broader endemic fluorosis project. The intervention period was 3 months in both regions. Because the predominant fluoride-exposure sources differed between the two endemic areas, the exposure-reduction strategies were source-specific rather than identical in content. In Jishan County, where water-borne fluorosis predominates, the intervention mainly consisted of drinking-water improvement measures, including the use of lower-fluoride water sources. In Ruoergai County, where brick-tea–borne fluorosis predominates, the intervention mainly focused on reducing brick-tea consumption, together with dietary health education aimed at lowering fluoride intake.

Urinary fluoride levels were measured before and after the three-month intervention period to assess recent fluoride exposure. Serum fluoride was not measured because it is more susceptible to short-term physiological, dietary, and sampling-related fluctuations, whereas urinary fluoride was considered more suitable for reflecting recent fluoride exposure in this field-based follow-up setting. Compliance was monitored by village physicians through telephone follow-up. In addition, approximately 10% of participants were randomly selected for adherence checks. These procedures were used to document intervention implementation and participant adherence during the three-month follow-up period.

### 2.4 Statistical analysis

All statistical analyses were conducted using SPSS version 28.0. Categorical variables (sex, joint ultrasound findings, DR findings) were compared between groups using the chi-square test. Continuous variables (age, IOM thickness) are expressed as mean ± standard deviation and were compared using the independent-samples t-test. Fluorosis grade distributions before and after intervention were compared using the chi-square test. A two-sided *p* value less than 0.05 was considered statistically significant. Categorical variables were summarized as counts and percentages. For key between-region comparisons, risk differences (RDs) with 95% confidence intervals (CIs) were calculated to indicate the magnitude and precision of observed differences. Continuous variables were expressed as mean ± standard deviation, and between-group differences were reported as mean differences (MDs) with 95% CIs. Detection rates for DR and MSKUS were reported with 95% CIs. The chi-square test was used for categorical comparisons when appropriate, and Fisher’s exact test was considered when expected cell counts were small. Continuous variables were compared using the independent-samples t-test. Detection rates of DR and MSKUS in the full cohort were summarized descriptively with 95% CIs. Because complete lesion-level paired cross-tabulated data were not available for all DR–MSKUS comparisons, no formal paired inferential test was performed for these modality comparisons. A two-sided *p* value less than 0.05 was considered statistically significant.

## 3 Results

### 3.1 Demographic characteristics

The mean age at diagnosis was higher in Jishan County patients than in Ruoergai patients (64.73 ± 7.73 years vs. 59.35 ± 9.52 years; MD: 5.38 years, 95% CI: 3.18–7.58; *p* < 0.05). The two cohorts also differed in sex distribution, with a relatively higher proportion of female patients in Jishan County. These baseline demographic differences should be considered when interpreting the observed regional differences in imaging findings.

### 3.2 Elbow joint ultrasound findings

Ruoergai patients demonstrated higher observed detection rates of humeroulnar joint calcification than Jishan County patients (78.07% vs. 15.15%; RD: 62.92 percentage points, 95% CI: 51.92–71.32), as well as humeroradial joint calcification (48.25% vs. 3.03%; RD: 45.22 percentage points, 95% CI: 35.16–54.45) and elbow joint effusion (32.46% vs. 0.76%; RD: 31.70 percentage points, 95% CI: 23.14–40.83; all *p* < 0.001; Table 1). Overall, elbow MSKUS findings differed between the two regional cohorts. Given the regional and demographic differences between groups, these findings should be interpreted cautiously and may reflect the combined influence of fluoride exposure pattern, regional background, and population-level characteristics. Elbow joint effusion was rarely observed in the Jishan County group but was detected in nearly one-third of patients in the Ruoergai group, suggesting more frequent intra-articular fluid accumulation in the latter cohort. By contrast, rates of common extensor tendon calcification (21.05% vs. 12.12%; *p* = 0.058) and common flexor tendon calcification (12.28% vs. 6.06%; *p* = 0.088) did not differ significantly between groups, suggesting that tendon calcification in skeletal fluorosis may follow a more uniform regional pattern less influenced by fluoride source.

**Table 1:** Elbow joint ultrasound findings in patients from both regions [n (%)].

Group	*n*	Humeroulnar Joint Calcification	Humeroradial Joint Calcification	Common Extensor Tendon Calcification	Common Flexor Tendon Calcification	UCL Calcification	Elbow Effusion
Jishan County	132	20 (15.15%)	4 (3.03%)	16 (12.12%)	8 (6.06%)	5 (3.79%)	1 (0.76%)
Ruoergai	114	89 (78.07%)	55 (48.25%)	24 (21.05%)	14 (12.28%)	2 (1.75%)	37 (32.46%)
RD, percentage points (95% CI)	–	62.92 (51.92–71.32)	45.22 (35.16–54.45)	8.93 (−0.42–18.44)	6.22 (−1.05–14.08)	−2.03 (−7.00–2.93)	31.70 (23.14–40.83)
*p* value	–	<0.001	<0.001	0.058	0.088	0.339	<0.001

UCL: ulnar collateral ligament. *p* values were derived from chi-square tests or Fisher’s exact tests, as appropriate.

### 3.3 Knee joint ultrasound findings

The prevalence of suprapatellar bursa effusion was significantly higher in Ruoergai patients than in Jishan County patients (39.47% vs. 12.12%; RD: 27.35 percentage points, 95% CI: 16.60–37.57; *p* < 0.001; Table 2), suggesting more frequent periarticular fluid accumulation in the Ruoergai cohort. In contrast, the observed detection rates of quadriceps tendon calcification, medial collateral ligament calcification, pes anserine tendon calcification, iliotibial band calcification, and patellar tendon calcification did not differ significantly between groups. Overall, compared with elbow joint findings, most knee periarticular calcification findings showed relatively similar distributions between the two regional cohorts.

**Table 2:** Knee joint ultrasound findings in patients from both regions [*n* (%)].

Group	*n*	Quadriceps tendon calcification	MCL calcification	Pes Anserine tendon calcification	ITB calcification	Patellar tendon calcification	Patellar ligament calcification	Suprapatellar bursa effusion
Jishan County	132	3 (2.27%)	1 (0.76%)	1 (0.76%)	0 (0.00%)	3 (2.27%)	0 (0.00%)	16 (12.12%)
Ruoergai	114	4 (3.51%)	4 (3.51%)	0 (0.00%)	1 (0.88%)	4 (3.51%)	3 (2.63%)	45 (39.47%)
RD, percentage points (95% CI)	–	1.24 (−3.45–6.61)	2.75 (−1.33–8.00)	−0.76 (−4.20–2.57)	0.88 (−2.04–4.79)	1.24 (−3.45–6.61)	2.63 (−0.72–7.49)	27.35 (16.60–37.57)
*p* value	–	0.561	0.127	0.352	0.281	0.561	0.061	<0.001

MCL, Medial collateral ligament; ITB, Iliotibial band. *p* values were derived from chi-square tests or Fisher’s exact tests, as appropriate.

### 3.4 Interosseous membrane thickness

The observed mean forearm IOM thickness in pronation was greater in the Ruoergai group than in the Jishan County group (2.02 ± 0.54 mm vs. 1.83 ± 0.49 mm; MD: 0.19 mm, 95% CI: 0.06–0.32; *p* = 0.004; Table 3). However, because different ultrasound systems and transducers were used in the two cohorts, this quantitative difference should be interpreted with caution. The observed difference may reflect disease-related variation, technical variability, or both. Leg IOM thickness at the mid-shaft showed only a small numerical difference between the two groups (1.21 ± 0.19 mm vs. 1.23 ± 0.16 mm; MD: −0.02 mm, 95% CI: −0.06 to 0.02; Table 3), and its clinical relevance appears limited.

**Table 3:** Interosseous membrane thickness comparison between both regions (mean ± SD, mm).

Group	*n*	Forearm IOM thickness in pronation (mm)	Leg IOM thickness at mid-shaft (mm)
Jishan County	132	1.83 ± 0.49	1.23 ± 0.16
Ruoergai	114	2.02 ± 0.54	1.21 ± 0.19
MD, mm (95% CI)	–	0.19 (0.06–0.32)	−0.02 (−0.06 to 0.02)
*p* value	–	0.004	0.377

IOM, Interosseous membrane. *p* values derived from independent-samples *t*-tests.

### 3.5 Changes in urinary fluoride levels and fluorosis severity grade after intervention

Among participants with paired urinary fluoride measurements, urinary fluoride levels decreased from 4.83 ± 3.21 mg/L at baseline to 3.91 ± 2.29 mg/L after intervention in the Jishan County group, and from 1.42 ± 0.92 mg/L to 1.02 ± 0.71 mg/L in the Ruoergai group. These findings suggest a reduction in recent fluoride exposure during the three-month intervention period. At baseline, 105 of 246 patients were classified as grade 1 (42.7%, 95% CI: 36.7–48.9), 134 as grade 2 (54.5%, 95% CI: 48.2–60.6), and 7 as grade 3 (2.8%, 95% CI: 1.4–5.8). Follow-up severity grading was available for 238 patients at the three-month follow-up after the exposure-reduction period. Among these patients, 114 were classified as grade 1 (47.9%, 95% CI: 41.6–54.2), 123 as grade 2 (51.7%, 95% CI: 45.4–58.0), and 1 as grade 3 (0.4%, 95% CI: 0.1–2.3). The distribution of severity grades differed significantly between baseline and the three-month follow-up (*p* < 0.05). However, given the relatively short follow-up interval and the absence of baseline MSKUS data, the clinical and structural implications of this change should be interpreted cautiously.

### 3.6 Comparison of DR and MSKUS detection rates

**Figure 2:**
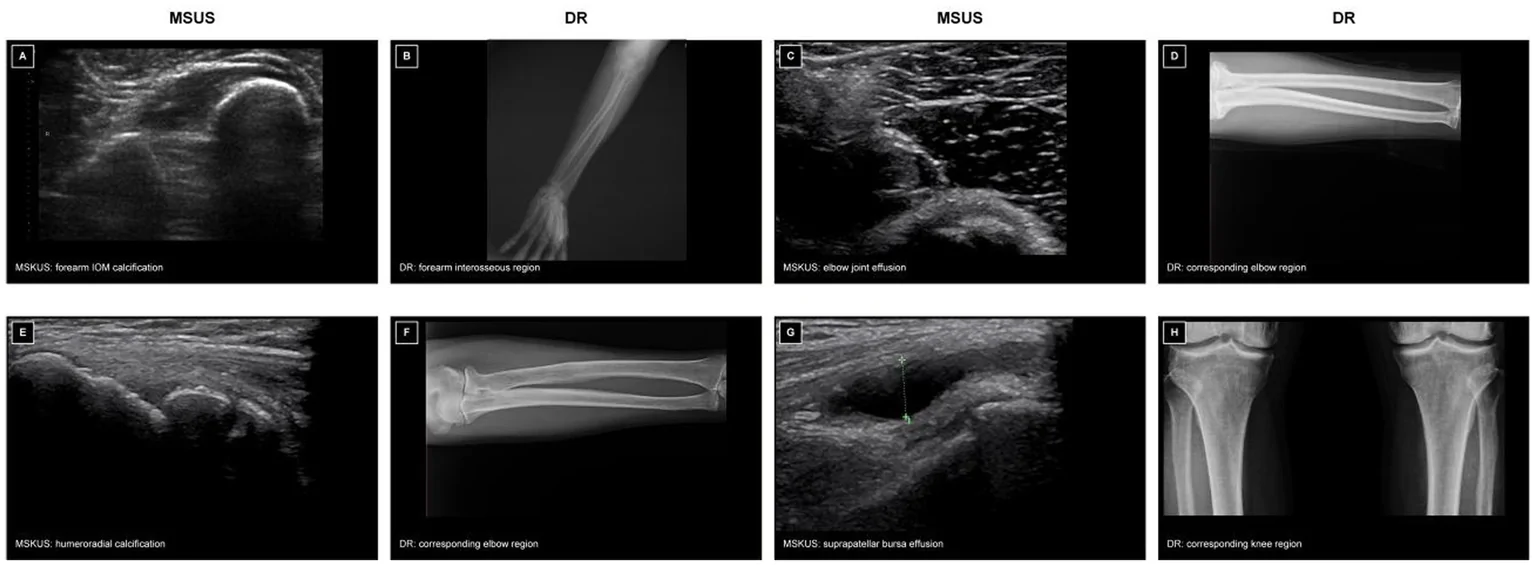
Representative musculoskeletal ultrasound and digital radiography findings in endemic skeletal fluorosis. **(A,B)** Musculoskeletal ultrasound (MSKUS) image showing calcification of the forearm interosseous membrane (IOM), with the corresponding digital radiography (DR) image of the forearm region. **(C,D)** MSKUS image showing elbow joint effusion, with the corresponding DR image of the elbow region. **(E,F)** MSKUS image showing humeroradial joint calcification, with the corresponding DR image of the elbow region. **(G,H)** MSKUS image showing suprapatellar bursa effusion, with the corresponding DR image of the knee region. MSKUS, musculoskeletal ultrasound; DR, digital radiography; IOM, interosseous membrane.

A descriptive comparison of DR and MSKUS findings across all 246 patients showed lesion-specific differences in observed detection patterns (Table 4). Representative MSKUS and corresponding DR findings are shown in Figure 2. DR identified radial crest enlargement in 232 patients (94.3%, 95% CI: 90.7–96.6), whereas MSKUS detected this finding in 123 patients (50.0%, 95% CI: 43.8–56.2). In contrast, MSKUS detected radius-ulna IOM calcification in 181 patients (73.6%, 95% CI: 67.7–78.7), whereas DR detected this finding in 39 patients (15.9%, 95% CI: 11.8–20.9). Detection rates for tibia-fibula IOM calcification were 9.3% (95% CI: 6.3–13.6) for DR and 7.3% (95% CI: 4.7–11.3) for MSKUS. Cortical loosening at the pronator attachment was identified by DR in 75 patients (30.5%, 95% CI: 25.1–36.5) but was not detected by MSKUS. Soleus tendon calcification was detected by DR in 62 patients (25.2%, 95% CI: 20.2–31.0) but was not detected by MSKUS. These findings suggest that DR and MSKUS provide complementary, non-redundant imaging information in endemic skeletal fluorosis.

**Table 4:** Comparison of DR and MSKUS detection rates in the full cohort (*n* = 246).

Modality	*n*	Radial crest enlargement	IOM calcification (Radius-Ulna)	IOM calcification (Tibia-Fibula)	Cortical loosening at pronator attachment	Soleus tendon calcification
DR n (%; 95% CI)	246	232 (94.3; 90.7–96.6)	39 (15.9; 11.8–20.9)	23 (9.3; 6.3–13.6)	75 (30.5; 25.1–36.5)	62 (25.2; 20.2–31.0)
MSKUS n (%; 95% CI)	246	123 (50.0; 43.8–56.2)	181 (73.6; 67.7–78.7)	18(7.3; 4.7–11.3)	0 (0.0; 0.0–1.5)	0 (0.0; 0.0–1.5)

Data are presented as *n* (%; 95% CI). DR, digital radiography; MSKUS, musculoskeletal ultrasound; IOM, interosseous membrane. Because complete lesion-level paired cross-tabulated data were not available for all DR–MSKUS comparisons, the modality comparison was performed descriptively without formal paired inferential testing.

## 4 Discussion

This study compared musculoskeletal ultrasound (MSKUS) and digital radiography (DR) findings in patients with endemic skeletal fluorosis from two endemic regions with different predominant fluoride-exposure patterns: Jishan County, where exposure was mainly water-borne, and Ruoergai, where exposure was mainly brick-tea-borne. Building on previous radiographic studies that have largely focused on osseous and mineralized structural abnormalities, this study further characterized MSKUS-detectable periarticular calcifications, joint effusions, and interosseous membrane (IOM) abnormalities. DR examinations were available at baseline and at the three-month follow-up, whereas MSKUS was performed only at the three-month follow-up. Therefore, the MSKUS findings should be interpreted as cross-sectional follow-up imaging phenotypes rather than longitudinal ultrasound changes. Prospective studies incorporating baseline and serial follow-up MSKUS examinations are needed to clarify temporal changes in ultrasound features.

Patients from Ruoergai showed higher observed detection rates of humeroulnar joint calcification, humeroradial joint calcification, elbow effusion, and suprapatellar bursa effusion than patients from Jishan County. These findings suggest that periarticular and superficial soft-tissue involvement may differ between the two regional cohorts. Previous studies have shown that chronic excessive fluoride exposure is associated with skeletal fluorosis and musculoskeletal involvement, including osteosclerosis, ligamentous calcification, joint stiffness, restricted mobility, and functional impairment (1, 14). In brick-tea-drinking populations, long-term consumption of fluoride-rich brick tea has been reported as an important source of fluoride exposure and has been associated with adult skeletal fluorosis in Tibetan areas (10, 11, 15). These observations provide a plausible epidemiological and clinical context for the more frequent periarticular abnormalities observed in the Ruoergai cohort. However, because this study did not directly measure brick-tea fluoride content, tea consumption volume, water fluoride concentration, nutritional status, occupational exposure, or cumulative fluoride dose, the between-region differences should be interpreted as regional imaging associations rather than causal evidence that fluoride source itself determined the ultrasound phenotype.

Age and sex differences may also have influenced the between-group imaging findings. Patients from Jishan County were older and included a higher proportion of female participants. Previous literature suggests that the clinical and radiographic manifestations of skeletal fluorosis may be affected by multiple host- and exposure-related factors, including age, dose and duration of fluoride exposure, nutritional status, calcium intake, dietary habits, and skeletal sites involved (1, 4). These demographic and regional factors should therefore be considered when interpreting the imaging findings. Although patients from Ruoergai were younger on average, they showed higher observed detection rates of several periarticular calcifications and effusions. This pattern may reflect combined effects of exposure pattern, regional background, lifestyle, nutritional status, and other unmeasured factors, rather than a single causal mechanism.

The higher observed detection rates of elbow effusion and suprapatellar bursa effusion in the Ruoergai cohort suggest that MSKUS can provide additional information on intra-articular or periarticular fluid accumulation that is not directly characterized by DR. Musculoskeletal ultrasound is commonly used to assess superficial soft-tissue disease, fluid collections, tendons, ligaments, joint recesses, synovium, and dynamic joint or tendon movement (7, 8). However, the biological basis of the effusion findings in this study remains uncertain. Because inflammatory laboratory markers, synovial pathological findings, and longitudinal MSKUS data were unavailable, these effusions should be interpreted as imaging observations rather than direct evidence of fluoride-related synovitis or active inflammation.

The IOM thickness findings are also notable but require cautious interpretation. Radiographic studies have shown that skeletal fluorosis may involve calcification or ossification of ligamentous, tendinous, muscular attachment sites, and interosseous membranes (4). In adult brick-tea-type skeletal fluorosis, interosseous membrane ossification, tendon attachment calcification, articular degeneration, arthropathy, and functional disability have also been described (11). These findings suggest that IOM abnormalities may represent a meaningful target for MSKUS assessment in endemic skeletal fluorosis. However, the absolute between-group differences in IOM thickness in the present study were small, particularly for leg IOM thickness. In addition, the two cohorts were examined using different ultrasound systems and transducers, without inter-equipment calibration, phantom-based reproducibility assessment, or cross-platform validation. Although standardized scanning procedures, fixed positioning, anatomical landmarks, predefined measurement sites, and repeated measurements were used, the IOM thickness results should be regarded as exploratory quantitative observations rather than definitive evidence of subtype-specific structural differences.

DR and MSKUS showed lesion-specific differences in observed detection patterns. Radiography is well suited for depicting mineralized skeletal and entheseal abnormalities in skeletal fluorosis, including osteosclerosis, skeletal deformity, ligamentous or tendinous ossification, and interosseous membrane ossification (4). In contrast, MSKUS is suitable for real-time evaluation of superficial periarticular soft tissues, tendons, ligaments, joint recesses, synovium, and fluid collections (6, 7). In this study, DR showed a higher observed detection rate for radial crest enlargement, whereas MSKUS showed a higher observed detection rate for radius-ulna IOM calcification. Because no independent reference standard, such as CT, MRI, or histopathology, was available, these findings should not be interpreted as evidence of superior diagnostic sensitivity or specificity. Instead, they indicate modality-specific differences in observed detection rates. Clinically, DR and MSKUS should be considered complementary rather than competing modalities: DR remains important for osseous assessment, whereas MSKUS may serve as an adjunctive modality for evaluating superficial soft-tissue calcifications, joint effusions, and IOM abnormalities.

The distribution of fluorosis severity grades changed after 3 months of fluoride-exposure reduction. Severity reclassification was primarily based on clinical assessment combined with DR findings according to WS/T 192–2021, and MSKUS findings were not used for grading. Reducing fluoride exposure remains fundamental for the prevention and management of endemic fluorosis (1, 16, 17). Nevertheless, skeletal fluorosis is a chronic disorder characterized by mineralized structural abnormalities, including osteosclerosis and ligamentous or tendinous calcification/ossification (1, 4). Substantial reversal of established skeletal or periarticular structural lesions within 3 months would therefore not generally be expected. In this study, the post-intervention change in severity-grade distribution should be interpreted cautiously and may reflect changes in symptoms, grading variability, baseline classification fluctuation, regression to the mean, or differences in follow-up availability rather than definite structural reversal. Because MSKUS was performed only at the three-month follow-up, this study mainly describes the ultrasound imaging phenotype at that time point. Future studies evaluating the monitoring role of MSKUS should include both baseline and serial follow-up ultrasound examinations.

The clinical relevance of this study lies in its regional comparison of MSKUS findings and its descriptive evaluation of the complementary roles of MSKUS and DR in endemic skeletal fluorosis. In endemic and resource-limited settings, MSKUS offers practical advantages, including absence of ionizing radiation, portability, repeatability, and real-time dynamic imaging (6, 7). These features may make MSKUS useful as an adjunctive imaging modality for assessing superficial soft-tissue calcifications, joint effusions, and IOM abnormalities. At this stage, however, MSKUS should not be considered a replacement for DR. Combining both modalities may improve the comprehensiveness of musculoskeletal imaging assessment in endemic skeletal fluorosis.

Several limitations should be acknowledged. First, the retrospective design may have introduced selection and information biases. Second, the two cohorts were recruited from different geographic regions, and the observed differences may have been influenced by demographic, environmental, nutritional, lifestyle-related, occupational, and healthcare-related factors. Third, MSKUS was performed only at the three-month follow-up, without baseline ultrasound data; therefore, this study describes follow-up ultrasound imaging phenotypes rather than longitudinal ultrasound changes. Fourth, different ultrasound systems and transducers were used between groups, and no inter-equipment calibration, phantom-based reproducibility assessment, or cross-platform validation was performed. Fifth, formal intra-observer and inter-observer reliability analyses were not conducted. Although standardized scanning procedures and repeated measurements were used, the operator-dependent nature of ultrasound remains relevant. Sixth, urinary fluoride was measured before and after intervention and showed a post-intervention decrease in both cohorts; however, water fluoride, serum fluoride, brick-tea fluoride content, tea consumption volume, nutritional indicators, cumulative fluoride exposure, and other potential confounders were not systematically assessed. Finally, because no independent reference standard was available, this study compared observed detection rates between DR and MSKUS rather than formal diagnostic performance.

Future prospective multicenter studies should include baseline and serial follow-up MSKUS examinations, harmonized or calibrated ultrasound equipment, and formal intra-observer, inter-observer, and inter-equipment reliability assessments. Integration of urinary fluoride, water fluoride, brick-tea fluoride content, tea consumption volume, nutritional indicators, and other environmental exposure data would help clarify the relative contributions of disease subtype, regional characteristics, and technical factors to imaging findings. Such standardized longitudinal evidence is needed to define the adjunctive role of MSKUS in screening, lesion characterization, and follow-up assessment of endemic skeletal fluorosis.

## 5 Conclusion

DR and MSKUS provided complementary imaging information in patients with endemic skeletal fluorosis. DR remained valuable for identifying osseous structural abnormalities, whereas MSKUS further demonstrated superficial soft-tissue calcifications, joint effusions, and IOM abnormalities. Differences in several ultrasound findings and IOM thickness measurements were observed between the Ruoergai and Jishan County cohorts, suggesting that musculoskeletal involvement may vary across endemic regions. Given the retrospective regional comparative design, single follow-up MSKUS assessment, differences in ultrasound equipment and transducers, lack of inter-equipment calibration and formal reproducibility assessment, and potential regional and demographic confounding, these findings should be interpreted as exploratory cohort-level findings. Prospective multicenter studies with standardized imaging protocols, calibrated ultrasound systems, and baseline plus serial follow-up MSKUS assessments are warranted to further validate the adjunctive value of MSKUS in comprehensive imaging evaluation and follow-up of endemic skeletal fluorosis.

## Data Availability

The original contributions presented in the study are included in the article. Further inquiries can be directed to the corresponding author.
